# Neural Correlates of Visual Aesthetics – Beauty as the Coalescence of Stimulus and Internal State

**DOI:** 10.1371/journal.pone.0031248

**Published:** 2012-02-22

**Authors:** Richard H. A. H. Jacobs, Remco Renken, Frans W. Cornelissen

**Affiliations:** 1 Laboratory for Experimental Ophthalmology, School for Behavioral and Cognitive Neurosciences, University Medical Center Groningen, University of Groningen, Groningen, the Netherlands; 2 BCN NeuroImaging Center, School for Behavioral and Cognitive Neurosciences, University Medical Center Groningen, University of Groningen, Groningen, the Netherlands; University of British Columbia, Canada

## Abstract

How do external stimuli and our internal state coalesce to create the distinctive aesthetic pleasures that give vibrance to human experience? Neuroaesthetics has so far focused on the neural correlates of observing beautiful stimuli compared to neutral or ugly stimuli, or on neural correlates of judging for beauty as opposed to other judgments. Our group questioned whether this approach is sufficient. In our view, a brain region that assesses beauty should show beauty-level-dependent activation during the beauty judgment task, but not during other, unrelated tasks. We therefore performed an fMRI experiment in which subjects judged visual textures for beauty, naturalness and roughness. Our focus was on finding brain activation related to the rated beauty level of the stimuli, which would take place exclusively during the beauty judgment. An initial whole-brain analysis did not reveal such interactions, yet a number of the regions showing main effects of the judgment task or the beauty level of stimuli were selectively sensitive to beauty level during the beauty task. Of the regions that were more active during beauty judgments than roughness judgments, the frontomedian cortex and the amygdala demonstrated the hypothesized interaction effect, while the posterior cingulate cortex did not. The latter region, which only showed a task effect, may play a supporting role in beauty assessments, such as attending to one's internal state rather than the external world. Most of the regions showing interaction effects of judgment and beauty level correspond to regions that have previously been implicated in aesthetics using different stimulus classes, but based on either task or beauty effects alone. The fact that we have now shown that task-stimulus interactions are also present during the aesthetic judgment of visual textures implies that these areas form a network that is specifically devoted to aesthetic assessment, irrespective of the stimulus type.

## Introduction

How do external stimuli and internal state coalesce to create the distinctive aesthetic pleasures that give vibrance to human experience? The answer to this question can be found in the brain, that delicate machine that assumes different states reflecting our moods and intentions, and that processes the information impinging on our senses. Neuroaesthetics research is concerned with the neural correlates of our aesthetic experiences, in particular the experience of beauty. Neuroimaging techniques make it possible to investigate brain activation associated with our processing of sensory information and with the ensuing experiences.

In recent years, several studies have been performed to investigate the neural correlates of human aesthetic experience. This research has taken two approaches: 1) The investigation of neural correlates of beauty level, i.e. brain regions that differentiate between beautiful and ugly stimuli that were presented to participants [1], [2], [3], [4], and 2) The investigation of beauty judgment as opposed to other judgments [5].

However, we believe the most interesting question in this type of research is the following: where does the beauty assessment actually take place? Is it where the brain differentiates between the different beauty levels? This is unlikely. Beauty judgments are to some extent predictable from the features that are present in the stimuli [6]. Because of this relationship between beauty and features, the observed brain activations may be caused by the processing of these features, rather than by the experience of beauty itself.

Studies examining the effects of stimulus beauty have reported many different brain regions, including the occipital and premotor cortex [1], the fusiform gyrus [2], the ventral tegmentum, the amygdala and the nucleus accumbens [3], and the orbitofrontal and motor cortex [4]. In the tactile domain (where pleasantness rather than beauty ratings were given), the orbitofrontal cortex, rostral anterior cingulate cortex, and amygdala [7] have been reported. These divergent findings may be explained by the processing of beauty-related features in the different stimuli, and may be less related to beauty aspects themselves.

Instead, we could compare the activations related to different judgments, which we believe is a more plausible approach. We would certainly expect a brain region involved in beauty assessment to be more active during the judgment of beauty than during other types of judgment. The posterior cingulate and frontomedian cortex have been reported to be more active during beauty judgments than during symmetry judgments [5]. But, we would also expect a beauty assessment to result in a response that differentiates between beautiful, neutral and ugly stimuli. In other words, we would expect an interaction between the type of judgment and beauty level in regions that are truly involved in making a beauty assessment. Brain regions merely differentiating between judgments – and not between beauty levels – may support the beauty assessment, but without actually performing the beauty assessment itself.

We identified three problems in the current literature on neuroaesthetics. The first is a focus on the main effects of judgment and of beauty level, which does not necessarily reflect the beauty assessment itself, as explained above. We believe that an enhanced activation to beautiful stimuli during beauty judgments, as compared to during other judgments about the same stimuli, provides much stronger evidence that a brain region is involved in assessing beauty.

The second problem is that the choice of the control task may influence the results. We found only a single study that focused on differences between judgments [5]. In this study, symmetry judgments were chosen as a baseline, on the premise that symmetric stimuli are usually judged to be more beautiful. Although the similarity of the tasks allows for interpreting the differences in brain activation as being specific for beauty judgments, it does not allow for interpretation in terms of the major factors influencing judgments. Semantic differential studies point to similarities between beauty judgments and other evaluative judgments, such as judgments of elegance, warmth, and interestingness [6], which as a group appear unrelated to a descriptive dimension, comprising judgments of roughness, complexity, and the age (of visually perceived textures). It seems desirable to first get a grip on these major factors, before zooming in on the subtle differences between the closely related judgments.

The third problem in the literature is that beauty level is confounded with features in the stimulus, as explained above. A related problem is that many different stimuli have been employed. The use of different stimulus types may explain the divergent findings, because of the different features in the stimuli, or because of the different associations people have with the complex stimuli, such as paintings.

To address the above issues, we designed an experiment in which we first varied both the beauty level of stimuli and the type of judgment, in a single paradigm. Second, we employed a control task that is sufficiently different from a beauty judgment to make sure that we are not factoring out some crucial common elements. To this end, we employed a roughness judgment, which has been shown in a semantic differential study to be orthogonal to beauty judgments [6]. From this latter study, it is clear that beauty judgments are representative for other evaluative judgments, while roughness judgments are representative for other more objective, or descriptive, judgments, such as judgments of age and complexity (textures were used as stimuli in this study, as well as in the present one). As a third judgment, we employed a naturalness judgments, because the semantic differential study showed that this type of judgement fell in-between the other judgments, being moderately related to both the evaluative and the descriptive judgment dimensions. Third, we employed visual textures as stimuli because they do not elicit many semantic associations. Moreover, these visual textures were individually selected for their beauty, so that the effects of beauty were enhanced. Due to individual differences in preferences, the effects of some (though probably not all) features would be levelled out. Fourth, by looking at interactions between beauty level and the type of judgment, we assumed that we would capture activity in brain regions involved in making beauty assessments per se, rather than in regions responding merely to features that happen to be associated with beauty, or in brain regions that merely support the making of beauty assessments, but without performing the actual assessment itself.

## Methods

### Participants

Ten men and eight women (age: 20–39), all right-handed, participated in the study. All had normal or corrected-to-normal visual acuity and gave their written informed consent according to procedures approved by the Medical Ethics Committee of the University Medical Center Groningen, the Netherlands, in accordance with the Declaration of Helsinki.

### Stimuli

**Figure 1:**
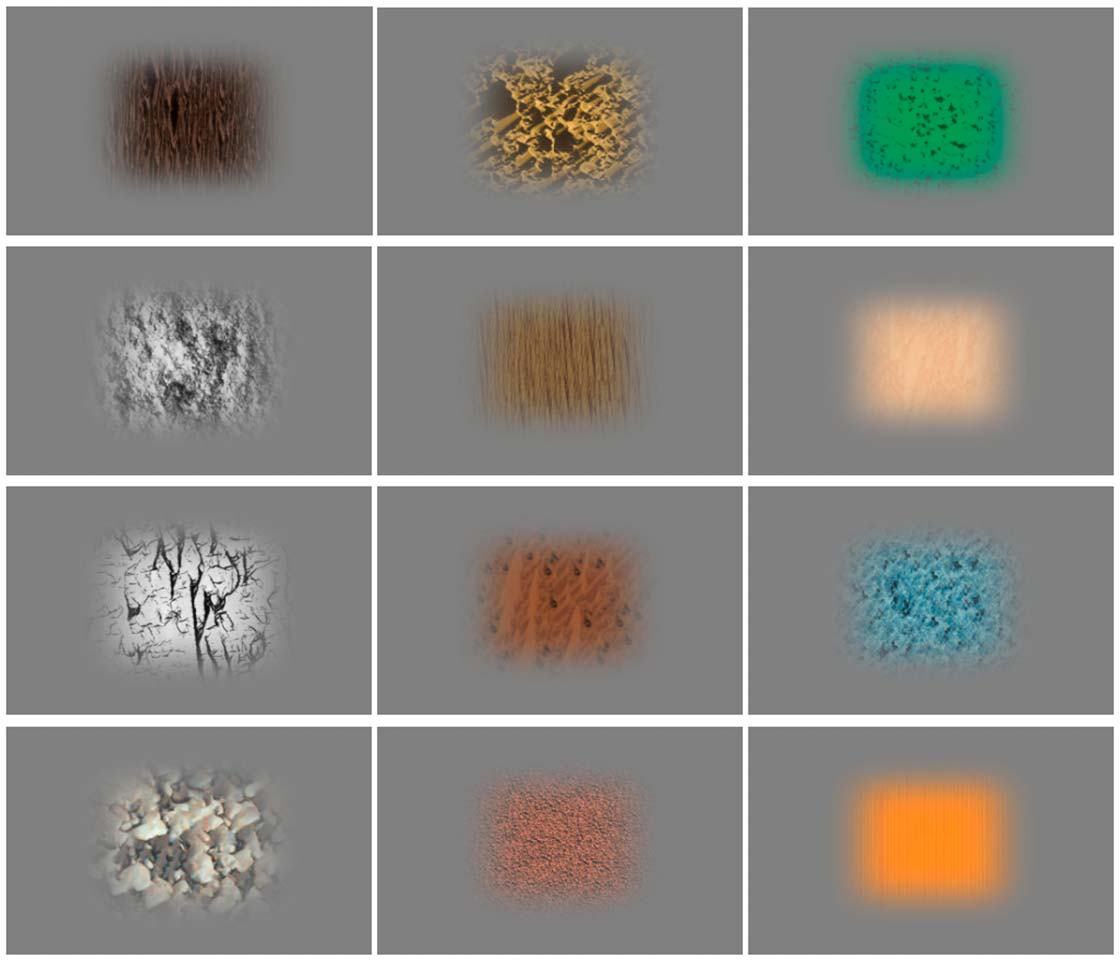
Example of textures used in the experiment. Textures were presented against a grey background. Computer-generated and photographed textures were used, some coloured and others in greyscale.

The stimuli were visual textures, which we defined as repetitive patterns in which no single object outline can be discerned. For current purposes, we take colour to be an integral part of textures for the following reasons: isoluminant colours can define textures, colour and texture are both surface properties, and previous neuroimaging experiments were not able to differentiate between texture and colour regions in the brain [8], [9]. It may be more appropriate to speak of surface properties than of textures, but we stick to the term ‘textures’ for brevity. We don't expect the distinction to be relevant for our findings and interpretations regarding beauty. Textures were collected from various internet sources (http://www.fundermax.at/, http://www.ux.uis.no/~tranden/brodatz.html, http://www.textureking.com/, http://inobscuro.com/textures/, http://textures.forrest.cz/). Stimulus sizes were standardized, using cropping to reduce the size of large textures, and a texture growth algorithm to enlarge small textures. Example of textures are shown in Figure 1.

### Stimulus presentation

In the initial texture selection procedure, stimuli were presented on a 30″ Apple Cinema HD Display monitor and were shown at a visual angle of about 22×22 degrees (viewing distance 70 cm), on a grey background (see Figure 1) with a mean luminance of 55 cd/m^2^.

In the fMRI scanner, stimuli were back-projected onto a translucent screen (44×34 cm) using a Barco LCD Projector G300 (Barco, Kortrijk, Belgium) set at a resolution of 800×600 pixels. The translucent screen subtended a visual angle of 32×25.5 degrees. Textures were presented at a size of about 13×13 degrees, on a grey background (see Figure 1) having a mean luminance of 3260 cd/m^2^. Stimuli were presented in Matlab (MathWorks, Natick, MA) with the Psychtoolbox (http://psychtoolbox.org/) extensions [10], [11] using an Apple Macbook Pro (Apple, Cupertino, CA).

### Texture selection

For each subject, the texture stimuli were selected from a collection of 436 textures based on a separate rating experiment. In this experiment, textures were presented one-by-one, and rated for beauty by moving a slider along a bar at the bottom of the screen. Based on the subject's judgment, the 12 textures judged least beautiful (negative valence) and the 12 judged most beautiful (positive valence) were selected, as well as 12 from the middle of the judgment range (neutral valence). These selected textures were used as stimuli in the functional magnetic resonance imaging (fMRI) experiment.

### fMRI-procedure

In the fMRI experiment, subjects performed three runs. During each run they judged textures for their beauty, naturalness and roughness. At the beginning of a run a fixation period lasting 30 s was presented.

Judgments were grouped into blocks of six trials and interleaved in pseudo-random order within a run. Within each judgment block, textures with positive, neutral and negative valence were presented in random order. During an entire run, each texture was presented only once for each judgment condition. Hence, during each run, all 36 textures were presented once for each type of judgment (for a total of 108 trials per run). Each texture was presented for 4000 ms (ISI = 1000 ms), during which the subject could indicate his or her judgment by pressing one of three buttons on a fibre-optics response pad (Current Designs Inc., Philadelphia, USA). Depending on the judgment condition, the buttons' meaning corresponded to beautiful, neutral and ugly (beauty judgment), or rough, neutral and smooth (roughness judgment), or natural, neutral and artificial (naturalness judgment). The words beautiful and ugly are antonyms, as has been empirically established [12]. Hence, they tap one perceptual dimension.

### Scanning parameters

The scanner was a 3T Philips Intera (Best, the Netherlands) with a sense-8 head coil. It was used to acquire T1 anatomical volume images (256×256 matrix, 160 slices, voxel size 1×1×1 mm) and T2*-weighted echo-planar images with blood oxygenation level-dependent contrast (64×64 matrix, voxel size 3.5×3.5×4 mm (no gap), TR = 2500 ms, TE = 28 ms, Field of View 224×224). Each echo-planar image consisted of 40 slices, acquired in descending order, positioned to cover the whole brain, except the cerebellum.

### Data analysis

Analysis was performed in BrainVoyager version 1.8 (Brain Innovation B.V., Maastricht, the Netherlands). A 2×2 model was specified at the individual level, with judgment conditions “beauty-roughness” and “beauty-naturalness”, and beauty levels “beautiful-neutral” and “beautiful-ugly”. Every trial was modelled as an event lasting 4 s. Activation levels of all runs were Z-transformed before 2^nd^ level analysis. A full-brain analysis was performed, with contrasts between the judgments and the beauty levels. Their interaction was also analysed. Activation to ugly-neutral stimuli and to roughness-naturalness judgments could be inferred by taking differences between the other contrasts.

Significance was thresholded at an alpha of 0.001 per voxel, with a minimum cluster size of 21 functional voxels (corresponding to 1029 mm^3^). This was done to achieve a corrected threshold of 0.05 for falsely reporting a positive result, as determined by the AlphaSim-tool (B.D. Ward, http://afni.nih.gov./afni/docpdf/AlphaSim.pdf).

In addition to this full-brain analysis, region-of-interest analyses were performed on the regions showing effects of task (beauty versus roughness) and beauty level (beautiful versus ugly), where we looked for interaction effects between task (specifically beauty judgments versus roughness judgments) and beauty level (beautiful versus ugly textures) within these regions at a significance threshold of 0.05.

## Results

The regions activated in our contrasts are shown in Table 1.

**Table 1:** Activation clusters in the contrasts between the judgments and the different beauty levels of the stimuli, and their interactions.

Contrast	Talairach coordinates	#Anatomical voxels	Brodmann Area	Region
beauty-roughness	−2, 63, 21	3043	10	frontomedian
beauty-roughness	−2, 57, 38	1863	9	frontomedian
beauty-roughness	−4, −49, 20	4806	30/31	posterior cingulate
roughness-beauty	−45, 4, 22	1204	44	frontal operculum
roughness-beauty	−50, −38, 40	5770	40	supramarginal gyrus
roughness-beauty	−56, −58, −7	1166	37	fusiform gyrus
positive-negative	−34, −44, −19	1924	cerebellum	culmen
positive-negative	−44, −75, −10	2708	18	visual cortex, middle occipital gyrus
Beauty (negative & neutral) & roughness (negative)>roughness (neutral)	−18, 24, −11		47	orbitofrontal cortex

The Talairach coordinates correspond to the centre-of-mass of the cluster. The cluster size is shown along with the Brodmann area and the name of the brain region.

### Contrasts between the judgments

**Figure 2:**
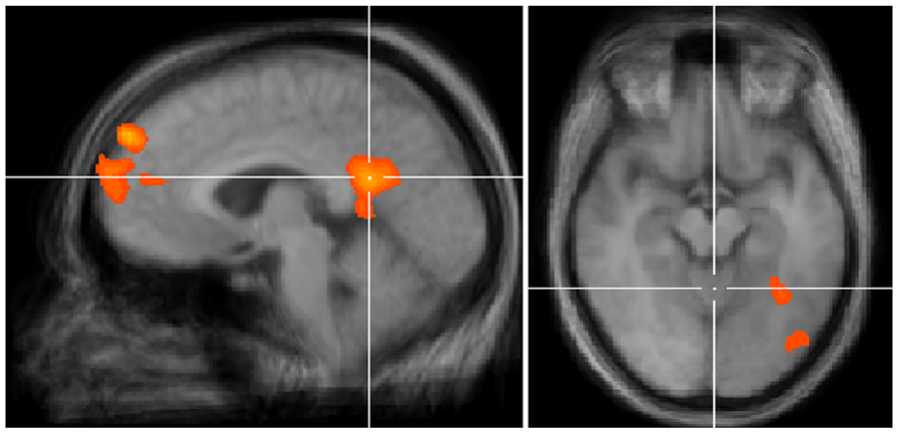
Examples of activation in the main contrasts. A. Comparison of activation during beauty versus roughness judgments shows signal increase in the frontomedian and posterior cingulate cortices. B. Comparison of activation for beautiful versus ugly stimuli shows signal increase in two clusters in the fusiform gyrus. Activation is presented for p<.001, uncorrected for multiple comparisons.

Regions that were activated more strongly during beauty judgments than during roughness judgments included the frontomedian cortex, the posterior cingulate cortex, and the amygdala (see Figure 2). Regions that were more active in the opposite contrast, roughness-beauty, involved the supramarginal gyrus, the frontal operculum and the fusiform gyrus. Activation related to naturalness judgments generally was not significantly different from either of the other judgments, with the exception of the supramarginal gyrus, where activation was lower for naturalness than for the other two judgments.

### Contrasts between beauty levels

The secondary visual cortex (Brodmann area 18/19; middle occipital and fusiform gyrus) was more active to positively valenced textures than to negatively valenced ones. No regions responded more strongly to the negatively valenced textures, and contrasts involving the neutral textures yielded no activations.

### Interactions between valence category and judgment condition

Our main interest was in finding and examining interactions between beauty levels (or the valence categories, namely beautiful, neutral, and ugly stimuli) and type of judgment (in particular beauty versus roughness judgments).

**Figure 3:**
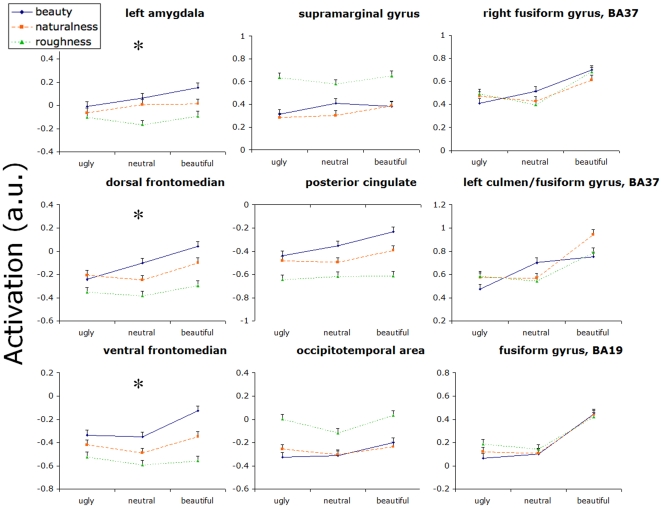
Activations in response to beautiful, neutral and ugly textures during beauty, naturalness and roughness judgments. Most regions-of-interest demonstrate significant interactions between beauty versus roughness judgments and beautiful (positive) versus ugly (negative) textures, as indicated by asterisks (*). Error bars indicate the standard error of the mean.

In the full-brain analysis, there were no brain regions displaying interaction effects at an uncorrected significance level of p<0.001. However, region-of-interest analyses in the regions displaying main effects of judgment showed that there were interaction effects in the amygdala (*t* = −2.260, *p* = 0.024) and the frontomedian cortex (ventral cluster *t* = −2.227, *p* = 0.026; dorsal cluster, *t* = −2.50, *p* = 0.012). Within the regions responding to beauty level, both the lateral (*t* = −2.707, *p* = 0.007) and the medial fusiform cluster (*t* = −2.899, *p* = 0.004) showed significant interaction effects. These effects are visualized in Figure 3, which also shows the effects of neutral stimuli and the naturalness judgments. It can be seen that the interactions between beauty level and type of judgment were qualitatively different for the regions responding to the main effect of judgment, when compared to the regions responding to the main effect of beauty level. The regions responding to the main effect of judgment were more responsive to beauty level during beauty judgments, and the differences were particularly pronounced for the beautiful stimuli. The regions responding to the effect of beauty level appeared rather to be less responsive to the ugly stimuli during the beauty judgment than during the other judgments. In addition, the activation of these regions to neutral stimuli was higher during beauty judgments than during the other judgments, while for positive stimuli the activation during beauty judgments could not be distinguished from that during the other two judgments. This pattern is hard to reconcile with a role in assessing the beauty of stimuli.

## Discussion

We were interested in brain activation related to the evaluative and descriptive judgment dimensions, as exemplified by beauty judgments (evaluative) and roughness judgments (descriptive), and related to differences in level of beauty. We were particularly interested in the interaction between judgment and beauty level, as this appeared to us to be the strongest indication that a region is truly involved in making a beauty assessment.

### Beauty levels

Contrasts between beautiful, neutral and ugly stimuli showed that the beautiful-ugly distinction was made only in two visual cortex clusters – regions that are distinct from the frontomedian and posterior cingulate cortices involved in making a beauty assessment. The coordinates (−44, −75, −10) of one of these clusters are an almost perfect match to those reported for preferred paintings in the fusiform gyri (−46, −74, −8, after conversion to Talairach coordinates) [2]. Since this is a visual region, its activation in response to beautiful stimuli may be a consequence of increased attention to the beautiful textures. If this were the case, one would expect the region to correspond to regions that are sensitive to the processing of textures. However, this region does not coincide with a region that was reported to increase activation when attention was directed at the texture, as opposed to the colour or form, of objects [9]. We conjecture that this region is responding to some low-level features in the textures that are associated with higher beauty ratings. For example, the presence of low spatial frequencies leads to higher beauty judgments [6]. Low frequencies are also characteristic of objects, as opposed to texture information, and the coordinates are close to those of a region that was more active when attention was directed at object shape [9].

### Evaluative brain regions

The contrast between beauty and roughness judgments should reveal the evaluative brain regions. Our study indicates that the frontomedian and the posterior cingulate cortex are evaluative regions. This finding agrees very well with previous studies comparing evaluative to non-evaluative judgments [5], [13]. This agreement is especially striking because these other studies have not based their choice of judgments on semantic differential findings. In fact, in one of these studies [13], the nature of the judgments (moral judgments) and the stimuli (sentences) was radically different from ours. This suggests a very general role for these regions in evaluative processing.

With regard to the posterior cingulate cortex, its role may not be the assessment of beauty itself. This is because interactions between judgment and beauty level – as described above for the orbitofrontal cortex – would have been indicative for such a role, yet such interactions were not found in this region. The posterior cingulate region may instead provide a more general supportive function, such as directing attention to one's inner world (i.e., self-reference), as opposed to the external world [14], [15], [16]. In fact, pleasantness ratings have been used to investigate internally cued, self-reference conditions [14]. The response of the frontomedian cortex, a brain region that often responds in a similar way as the posterior cingulate cortex, was sensitive to the interaction between beauty versus roughness judgments and beautiful versus ugly stimuli. This interaction pattern suggests that this region may truly be involved in assessing beauty, and even evaluative aspects in general.

### Descriptive brain regions

We assumed that the contrast between roughness and beauty judgments would reveal the regions that are recruited when making descriptive judgments. Our results indicated that these regions are the frontal operculum, the supramarginal gyrus, and the fusiform gyrus. However, the supramarginal gyrus did not meet the additional requirement that the naturalness judgment showed an intermediate activation strength. In most regions, naturalness did not differ significantly from either of the other judgments, but in the supramarginal gyrus, naturalness judgments were associated with significantly less activation than the other two judgments. Hence, we conclude that the frontal operculum and the fusiform gyrus remain candidate brain regions for the processing of descriptive judgments.

The supramarginal gyrus is generally recognized as belonging to secondary somatosensory cortex. As such it may not be surprising that it is involved in making roughness assessments. But its involvement in making roughness assessments of visually presented textures is rather surprising. Previous studies have reported visual cortical areas engaging in the analysis of tactile stimuli [17], [18], [19], [20], [21], [22], but to our knowledge the present study is the first report of a tactile region that is engaged in the analysis of visual stimuli. We conjecture that this activation occurs when subjects imagine touching the visually presented stimulus or the roughness sensations associated with it. However, this does not explain why activation during a naturalness judgment should be lower than during a beauty judgment. Figure 3 indicates that this is much smaller than the difference between roughness and the other judgments, so we should not place too much importance on the difference between beauty and naturalness judgments in this region.

### Interactions between beauty level and judgment type

Interactions between judgment type and beauty level point to beauty assessments resulting in beauty outcomes. A full-brain analysis did not highlight any regions displaying such interaction effects. Region-of-interest analyses on the clusters that appeared in the main effects of beauty-versus-roughness judgments and beautiful-versus-ugly stimuli indicated that many of these regions indeed show interaction effects between these two contrasts. This means that these clusters may be involved in beauty assessments. The fusiform gyrus, the amygdala and the frontomedian cortex all showed interaction effects. For the amygdala and the frontomedian cortex, these interactions consisted of stronger responses to beauty level during the judgment of beauty. This pattern is highly supportive for a direct involvement in beauty assessments.

In line with the amygdala finding, the only other study that looked at both beauty judgment and beauty level [23] reported that the right amygdala is more active to beautiful stimuli under explicit evaluation conditions. One other study reported that amygdala activation increased both in response to positive names (e.g. Mother Teresa) when subjects evaluated positive aspects of famous people, and to negative names (e.g. Adolf Hitler) when they evaluated negative aspects of famous people [24]. These findings provide further support for our contention that the amygdala is involved in making beauty assessments as well as more generally, evaluative assessments. In consideration of the amygdalar role in guiding selective attention (see Adolphs [25]), we believe that the amygdalar role in assessing beauty may consist of guiding attention to the features that are relevant for making the beauty or other evaluative assessments.

### The semantic differential as a basis for functional neuro-imaging

If the other studies investigating evaluative judgments had employed semantic differential studies on their judgments, they might have found their judgments to be orthogonal. We believe that showing the distinctness of the judgments empirically adds to the interpretability of the findings. It also predicts generalizability over other judgments loading on the same components, such as warmth, interestingness and colourfulness for the evaluative dimension, even though such generalizability remains to be demonstrated. In fact, Jacobsen et al. [5] may have chosen judgments that load on the same component, as they chose their symmetry and beauty judgment because they were correlated, and correlated judgments are likely to load on the same component. This highlights a way of further validating the semantic differential basis for distinguishing evaluative from non-evaluative processing, and distinguishing it from less empirically based approaches: There are cases in which the semantic differential studies point to a judgment, such as colourfulness, as being evaluative in nature [6], even though this is contrary to intuition and the assumptions in the other approaches. It would be interesting to see if colourfulness judgments would indeed be associated with the brain activation patterns similar to those found for beauty and other evaluative judgments, as predicted by the semantic differential approach, or rather to roughness and other non-evaluative judgments, as other approaches would predict.

Another prediction of the semantic differential approach would be that naturalness judgments should lead to activations intermediate between beauty and roughness judgments; semantic differential studies show that naturalness judgments fall in between these other two judgments in judgment space [6]. Although we generally did not find significant differences between naturalness and the other judgments in our whole-brain analysis, within our regions-of-interest, naturalness consistently fell in between the other judgments (see Figure 3). This suggests that the brain activations in these regions rather closely followed the pattern of the semantic differential studies.

### Limitations of this study

We used visual textures to investigate beauty assessment in the brain while minimizing semantic factors that are likely to play a role when faces, objects, or realistic paintings are used as stimuli. However, these stimuli may also be expected to lead to relatively shallow aesthetic reactions. Consequently, one may argue that we are not really assessing beauty reactions, but rather something related, such as liking or preference. While there are probably subtle distinctions between these concepts, we would expect these concepts to be strongly interrelated, and our prediction would be that they will all lead to similar reactions in the brain. In addition to using “shallow” stimuli, we presented stimuli for 4 seconds, which may be brief for a full-blown aesthetic reaction. It is quite possible that longer presentation times would have lead to different activation patterns, associated with a deeper processing of the stimuli. Indeed, Ishizu and Zeki [26] recently found that the orbitofrontal cortex was involved in assessing beauty irrespective of stimulus category, but its activation started to differ significantly only after observers viewed a stimulus for more than 4 seconds. Another limitation of our study is that we asked participants to rate the stimuli for beauty, but not for roughness or naturalness, before the study. This might have influenced the results. For example, it is possible that during scanning, participants remembered (or tried to remember) what response they had given before the scanning, in order to appear consistent. Supporting this possibility, Hofel and Jacobsen [27] claim that participants in their experiments wished to maintain consistency in their ratings. Hence, mnemonic factors may have played a role during the beauty, but not the roughness and naturalness judgments. In addition, the pre-scan ratings on beauty may have revealed to the participants that beauty was the core interest of the study, and it is possible that this influenced them in some way, e.g. by being more engaged in the beauty judgments than during the other judgments. Although this criticism is legitimate, the brain regions we report have also been found in previous neuro-aesthetics studies which did not ask participants to rate stimuli for beauty prior to the scanning. So, we believe that mnemonic factors and a possible awareness of the purpose of the study did not have a major impact on our findings.

Finally, there may be concerns regarding a possible circularity in the analysis [28], [29], [30]. We selected brain regions based on the main effects of task and stimulus valence. Within these selected brain regions, we looked for interaction effects between task and stimulus valence. The results of such a selection procedure would be inflated if the presence of interaction effects were dependent on the presence of main effects. However, interaction effects are statistically independent of the presence of main effects. Graphically, it can be seen that the presence of an interaction (e.g., smaller differences in activation between beauty and roughness judgments for beautiful stimuli than for ugly stimuli, i.e., converging lines), is not restricted by the presence of main effects (e.g., activation for beauty judgments on average being above activation for roughness judgments). Hence, concerns about a circularity in the analysis are not justified.

### Conclusions

We used semantic differential studies as an empirical basis for distinguishing between evaluative and descriptive judgments. We looked for brain regions responding to this distinction between judgments. We chose beauty as a representative judgment for the evaluative judgments, and roughness as a representative for the descriptive judgments. Besides the effects of judgment, we also looked at the effects of beauty level, and in particular at its interaction with the type of judgment. The frontomedian cortex and the amygdala appear to be selectively sensitive to beauty level during beauty judgments. Hence, these regions seem to compute a beauty outcome when attending to beauty, and may be directly involved in making beauty assessments. The fusiform gyrus was also sensitive to interactions between beauty level and type of judgment, but the pattern of these interactions is not commensurate with involvement in beauty assessments. The posterior cingulate cortex did not show an interaction with beauty level. Hence, this region appears to not be directly involved in making a beauty assessment itself. It may instead fulfil a supporting role, such as directing attention to the internal rather than the external world.

The frontal operculum and occipitotemporal area appeared responsive to the descriptive judgments, and may be directly or indirectly involved in making such judgments. These findings demonstrate the neural underpinnings of the judgment semantics. Another part of the fusiform gyrus distinguished between different beauty levels, but does not appear to make beauty assessments by itself.

By focusing on the interaction between beauty level and beauty judgments versus other judgments, we have narrowed down the regions that are potentially involved in making beauty assessments to the frontomedian cortex and the amygdala.
